# Realizing High Brightness Quasi‐2D Perovskite Light‐Emitting Diodes with Reduced Efficiency Roll‐Off via Multifunctional Interface Engineering

**DOI:** 10.1002/advs.202302232

**Published:** 2023-07-03

**Authors:** Yu‐Kuan Lin, Chiung‐Han Chen, Yen‐Yu Wang, Ming‐Hsuan Yu, Jing‐Wei Yang, I‐Chih Ni, Bi‐Hsuan Lin, Ivan S. Zhidkov, Ernst Z. Kurmaev, Yu‐Jung Lu, Chu‐Chen Chueh

**Affiliations:** ^1^ Department of Chemical Engineering National Taiwan University Taipei 10617 Taiwan; ^2^ Research Center for Applied Sciences Academia Sinica Taipei 11529 Taiwan; ^3^ Graduate Institute of Photonics and Optoelectronics National Taiwan University Taipei 10617 Taiwan; ^4^ National Synchrotron Radiation Research Center Hsinchu 30076 Taiwan; ^5^ Institute of Physics and Technology Ural Federal University Yekaterinburg 620002 Russia; ^6^ M. N. Mikheev Institute of Metal Physics of Ural Branch of Russian Academy of Sciences Yekaterinburg 620108 Russia; ^7^ Department of Physics National Taiwan University Taipei 10617 Taiwan; ^8^ Advanced Research Center for Green Materials Science and Technology National Taiwan University Taipei 10617 Taiwan

**Keywords:** efficiency roll‐off, hole blocking, interface engineering, Light‐emitting diodes, quasi‐2D perovskites

## Abstract

Quasi‐2D perovskites have recently flourished in the field of luminescence due to the quantum‐confinement effect and the efficient energy transfer between different *n* phases resulting in exceptional optical properties. However, owing to the lower conductivity and poor charge injection, quasi‐2D perovskite light‐emitting diodes (PeLEDs) typically suffer from low brightness and high‐efficiency roll‐off at high current densities compared to 3D perovskite‐based PeLEDs, which is undoubtedly one of the most critical issues in this field. In this work, quasi‐2D PeLEDs with high brightness, reduced trap density, and low‐efficiency roll‐off are successfully demonstrated by introducing a thin layer of conductive phosphine oxide at the perovskite/electron transport layer interface. The results surprisingly show that this additional layer does not improve the energy transfer between multiple quasi‐2D phases in the perovskite film, but purely improves the electronic properties of the perovskite interface. On the one hand, it passivates the surface defects of the perovskite film; on the other hand, it promotes electron injection and prevents hole leakage across this interface. As a result, the modified quasi‐2D pure Cs‐based device shows a maximum brightness of > 70,000 cd m^−2^ (twice that of the control device), a maximum external quantum efficiency (EQE) of > 10% and a much lower efficiency roll‐off at high bias voltages.

## Graphical abstract

A straightforward strategy for improving brightness and suppressing efficiency roll‐off in quasi‐2D perovskite light‐emitting diodes through perovskite/electron transporting layer (ETL) interface engineering is demonstrated. Modified devices with improved electronic properties show better performance at high bias voltages due to a more favorable energy level arrangement at the perovskite/ETL interface.

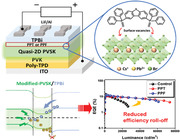

## 1 Introduction

In recent years, metal halide perovskites have attracted worldwide attention and have become one of the most promising optoelectronic materials due to their tunable semiconductor properties and processability in solution.^[^ ^1^, ^2^, ^3^, ^4^ ^]^ Among these, quasi‐2D perovskites, such as PEA_2_Cs_n‐1_Pb_n_Br_3n+1_, are particularly promising as luminescent materials due to their good stability and quantum confinement effects.^[^ ^5^, ^6^, ^7^, ^8^, ^9^, ^10^, ^11^, ^12^ ^]^ Furthermore, efficient carrier funnels derived from different quasi‐2D phases further improve the photoluminescence efficiency of quasi‐2D perovskites, making them even more attractive as luminescent materials.^[^ ^13^, ^14^, ^15^ ^]^ To date, quasi‐2D perovskite green light‐emitting diodes (PeLEDs) have achieved a record external quantum efficiency of 28%,^[^ ^16^ ^]^ demonstrating their excellent potential for luminescence applications. It is worth highlighting that by adjusting the distribution and/or composition of different quasi‐2D phases, one can also easily fabricate PeLEDs with different emission wavelengths, greatly reducing the difficulty of realizing efficient PeLEDs with different emission colors.^[^ ^17^, ^18^ ^]^

However, there is still room for improvement in quasi‐2D PeLEDs. Solution‐processed perovskite films typically have high defect concentrations and trap densities on the film surface, which can lead to severe non‐radiative recombination and thus degrade the performance of PeLEDs. Fortunately, surface passivation has been shown to be an effective method for reducing surface defects and suppressing non‐radiative recombination.^[^ ^19^, ^20^, ^21^, ^22^ ^]^ For example, phosphine oxide has become one of the most commonly used surface passivators for PeLEDs due to the strong electron‐donating properties of the P=O bond.^[^ ^23^, ^24^, ^25^, ^26^, ^27^, ^28^ ^]^ However, most of the phosphine oxides used in the literature to passivate perovskite surfaces are electrically insulating, which will inevitably affect carrier transport between the perovskite layer and the carrier transport layer.^[^ ^23^, ^24^, ^28^ ^]^ On the other hand, quasi‐2D perovskites typically show lower conductivity and poorer charge injection than 3D perovskites due to the insulating nature of the spacers. These drawbacks potentially lead to low brightness and severe efficiency roll‐off of quasi‐2D PeLEDs at high bias voltages and current densities,^[^ ^29^ ^]^ which will undoubtedly limit their widespread applications and commercialization.^[^ ^30^, ^31^ ^]^ Given this problem, the fabrication of efficient quasi‐2D PeLEDs with high brightness and low‐efficiency roll‐off is undoubtedly one of the most critical issues in this field.

To date, controlling the ratio between multiple quantum wells has been reported to facilitate cascade energy transfer and reduce the local carrier density in the quasi‐2D domains.^[^ ^32^ ^]^ As a result, unwanted non‐radiative recombination and efficiency roll‐off occurring at higher applied voltages can be considerably suppressed. In addition to this, the introduction of a barrier‐free charge injection layer can also help to reduce efficiency roll‐off by alleviating charge imbalance in the emitting layer.^[^ ^33^ ^]^ Based on these principles, we are interested in introducing an additional conductive phosphine oxide layer into the quasi‐2D PeLED to see how effective it is in reducing efficiency roll‐off. This is because the conductive phosphine oxide is not only able to passivate the defects in the perovskite layer,^[^ ^34^ ^]^ but is also conductive and has suitable energy levels to further modulate electron injection.^[^ ^35^, ^36^, ^37^, ^38^ ^]^

Hence, in this work, we introduce two conductive phosphine oxides, 2,8‐bis(diphenyl‐phosphoryl)‐dibenzo[b,d]thiophene (PPT) and 2,8‐bis(diphenyl‐phosphoryl)‐dibenzo[b,d]furan (PPF), at the interface between the perovskite layer and the electron transport layer (ETL). Thanks to phosphine oxide functional group, both PPT and PPF successfully passivate perovskite surface defects and inhibit non‐radiative recombination. Furthermore, due to their suitable energy levels, they act as electron transport materials at the perovskite/ETL interface, facilitating the injection of electrons into the perovskite layer while preventing hole leakage from the perovskite layer.^[^ ^35^, ^36^, ^37^, ^38^ ^]^ Improving the electronic properties at this corresponding interface is of considerable importance, as transient absorption (TA) spectroscopy shows that the modification of PPT/PPF does not alter energy transfer between multiple quasi‐2D phases in the perovskite film. Benefitting from these advantages, the performance of the modified devices is greatly improved, achieving a maximum brightness of 73,897 cd m^−2^, a maximum external quantum efficiency (EQE) of > 10% and a much lower efficiency roll‐off at high bias voltages, which is remarkable among the devices made from the same composition. The largely suppressed efficiency roll‐off of these modified devices demonstrates a straightforward and effective way to overcome the most challenging problems of quasi‐2D PeLEDs.

## 2 Results and Discussion

### 2.1 Modification of Quasi‐2D Perovskites by PPT/PPF Post‐Treatment

**Figure 1:**
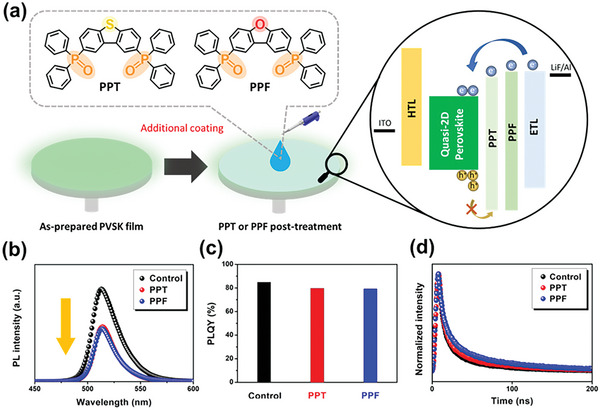
a) Chemical structures of PPT and PPF and schematic representation of the post‐treatment process and illustration of the interfacial engineering. b) PL emission spectra, c) PLQYs, and d) TRPL curves of pristine, PPT‐treated and PPF‐treated perovskite films, where the PLQYs were measured by a 368 nm laser.

As mentioned previously, we are interested in introducing conductive phosphine oxides to passivate the quasi‐2D perovskites and exploring their effectiveness in reducing efficiency roll‐off. Given the decent conductivity and suitable energy levels, we herein chose two phosphine oxides, PPT and PPF, and introduce them to the perovskite/ETL interface.^[^ ^39^, ^40^ ^]^ We hope that this additional layer will improve the charge injection at this corresponding interface as a result of cascade charge transfer, as shown in **Figure** **1**a. On the other hand, the P=O bond in phosphine oxides has been proven to passivate surface defects in the perovskite emitting layer, suppressing non‐radiative recombination, and enabling high‐performance light‐emitting devices. To proceed this, we herein spin‐coat an additional layer of PPT or PPF on the perovskite film prior to the ETL deposition, rather than introducing a simple post‐treatment by dissolving it in a washing solvent for dripping as reported in the literature. Note that in order to strengthen the passivation effect of PPT/PPF, the as‐prepared perovskite layer is soaked in the precursor solutions of PPT/PPF for a specific time prior to spin coating,^[^ ^23^ ^]^ and the details are described in the Experimental Section.

The introduction of phosphine oxide into perovskite precursors has been shown to influence the crystallinity and phase distribution of the derived quasi‐2D perovskite films.^[^ ^34^, ^41^ ^]^ Therefore, we first investigated the changes in crystallinity, optoelectronic properties, and film morphology of PPT/PPF‐modified perovskite films. Figure S1 (Supporting Information) displays the XRD characteristics of the pristine, PPT‐modified, and PPF‐modified perovskite films. As shown, there is no obvious difference in peak intensity or its position between these films. This indicates that neither crystallinity nor the distribution of the quasi‐2D phase is affected by the PPT/PPF modification. The Ultraviolet−visible (UV−Vis) absorption spectra of these films are present in Figure S2 (Supporting Information). In addition to showing similar absorption profiles, none of them show conspicuous absorption peaks for the small *n* phases (*n* < 5), indicating the suppression of low *n* phases in these films. Together with the XRD results, this indicates that the PPT/PPF modification does not alter the phase distribution and film quality of the quasi‐2D perovskite films.

It should be noted that PPT and PPF have been frequently used as the electron transport layers in organic light‐emitting diodes due to their good ability to transport electrons.^[^ ^35^, ^36^, ^37^, ^38^ ^]^ Therefore, in order to understand the influence of the PPT/PPF modification on the charge dynamics of these films, we performed the steady‐state photoluminescence (PL) spectroscopy and time‐resolved photoluminescence (TRPL) measurements. As presented in Figure 1b, the steady‐state PL spectra of both modified films display a clear PL quenching. This result reveals the occurrence of charge transfer between the perovskite layer and the PPT/PPF layer due to the good electron transport properties of both molecules and their suitable energy levels, as will be discussed later.^[^ ^39^, ^42^ ^]^ We also observed similar results from the photoluminescence quantum yields (PLQYs) of perovskite films, as shown in Figure 1c. We finally performed TRPL measurements to investigate the charge recombination behavior of these perovskite films (Figure 1d) and the biexponential fitting results are provided in Table S1 (Supporting Information). It can be seen that both modified films displayed a longer average carrier lifetime compared to the pristine film. This result suggests that, despite quenching the emission, both molecules are able to passivate the perovskite films. By passivating the surface defects, the non‐radiative recombination of the perovskite layer is suppressed, leading to an increase in the carrier lifetimes.^[^ ^43^ ^]^ Detailed interactions between perovskite and PPT/PPF are discussed in the next section.

We then examined the morphology of these films by scanning electron microscopy (SEM). Figure S3a (Supporting Information) presents the morphology of the pristine perovskite film, which shows a smooth and homogeneous surface. In order to exclude possible recrystallization effects caused by the solvent of the PPT/PPF precursor solution, we also examined the morphology of the film treated by pure chlorobenzene (CB). As shown in Figure S3b (Supporting Information), it showed a morphology comparable to the original film, which means that the treatment itself does not influence the perovskite surface if there is no PPT or PPF. Figure S3c,d (Supporting Information) displays the morphology of the PPT/PPF‐modified perovskite films. As shown, there are some nanoplatelets present on the film surface. These nanoplatelets probably originated from the aggregation of PPT/PPF. Despite the presence of these nanoplatelets, the morphology of both films remained similar to the pristine film and did not show any significant changes.

### 2.2 Interaction between PPT/PPF and Perovskite

**Figure 2:**
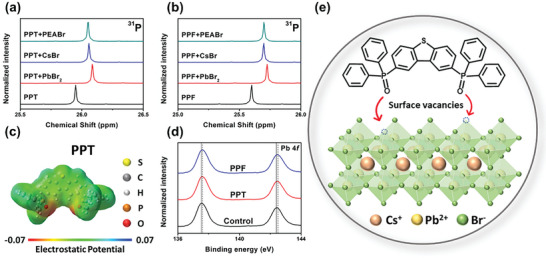
^31^P NMR spectra of a) PPT and b) PPF and their mixtures with different perovskite precursor components. c) ESP map of PPT molecule. d) XPS spectra of Pb 4f signal for the pristine, PPT‐modified, and PPF‐modified perovskite films. e) Schematic illustration of the passivation function of PPT on a perovskite surface.

In order to identify the interactions between PPT/PPF and perovskite, we investigated them by nuclear magnetic resonance (NMR), electrostatic potential (ESP) map and X‐ray photoelectron spectroscopy (XPS). As shown in the ^31^P NMR spectra (**Figure** **2**a,b), the original peak of phosphorous for pure PPT is at 25.9 ppm and shifts to ≈26.1 ppm after the introduction of PbBr_2_, CsBr, or PEABr. Similarly, the peak of pure PPF shifts from 25.6 to ≈25.7 ppm after mixing with the perovskite precursors. The difference in chemical shifts implies that the introduction of the perovskite precursors alters the chemical environment of the phosphorous nuclei, which is caused by coordination interactions between the P=O group and the cation.^[^ ^27^ ^]^ We then calculated the ESPs of PPT and PPF based on density functional theory to more accurately assess their interactions with perovskite. As shown in Figure 2c and Figure S4 (Supporting Information), it is obvious that the electron densities of PPT and PPF are predominantly located at the P=O group, giving them a strong Lewis basicity to coordinate with the uncoordinated Pb^2+^ ion.^[^ ^44^ ^]^ XPS also shows similar results. From the characteristic peak of P 2p shown in Figure S5 (Supporting Information), we clearly observe the presence of phosphorous on the perovskite surface, which indicates the successful formation of a PPT/PPF layer after post‐treatment. This evidence is consistent with the PL and SEM results. Furthermore, compared to the control film, the characteristic peak of Pb 4f in the PPT/PPF‐modified films shifted toward a higher binding energy (Figure 2d). This suggests a stabilizing effect of the P=O group on the surface of PbBr_6_^4−^ octahedral unit, which plays a major role in surface passivation.^[^ ^45^ ^]^ To sum up, from the ^31^P NMR and XPS results, we conclude that a PPT/PPF layer is successfully spin‐coated onto the perovskite film after post‐treatment and that the P=O groups of phosphine oxides thereby coordinate with surface defects and vacancies, as portrayed in Figure 2e. Moreover, due to the preferable coordination between the P = O group and the Pb^2+^ ion, the passivation effect primarily stems from the coordination between them, which is consistent with the reported literatures.

### 2.3 Influence of PPT/PPF Modification on Device Performance

**Figure 3:**
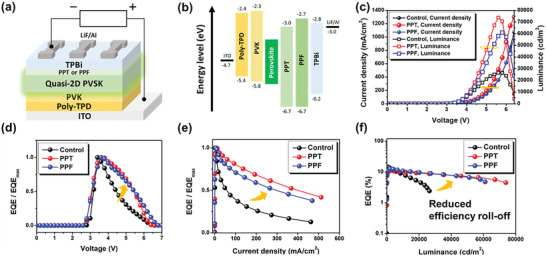
a) Structure and b) the energy‐level diagram of the fabricated PeLEDs. c) *J*−*V*−*L* characteristics, d) normalized EQE‐voltage curves, e) normalized EQE‐current density curves and f) EQE‐luminance curves of the fabricated devices.

Based on the above understanding, we then fabricate PeLEDs based on PPT/PPF‐modified perovskite films and compared their performance with the pristine film. The device structure employed in this work is ITO/poly‐TPD/PVK/quasi‐2D perovskite/TPBi/LiF/Al, as shown in **Figure** **3**a. As highlighted earlier, in the case of PPT/PPF‐modification, a PPT/PPF layer is introduced at the perovskite/TPBi interface and not just surface decoration on the perovskite film.

Figure 3b displays the detailed energy level diagram of our fabricated devices. As shown, both PPT and PPF help to prevent hole leakage from the perovskite layer to ETL, as their highest occupied molecular orbital (HOMO) levels are deeper than that of TPBi.^[^ ^46^ ^]^ To confirm this advantage, we fabricated the hole‐only devices with a configuration of ITO/poly‐TPD/PVK/quasi‐2D perovskite/MoO_3_/Ag based on the pristine, PPT‐ and PPF‐modified perovskite films. Figure S6 (Supporting Information) clearly shows that in PPT/PPF‐modified hole‐only devices, the hole‐dominated current density is significantly reduced, indicating that in these devices, the transport resistance is enhanced due to the hole‐blocking effected exerted by PPT and PPF. Furthermore, according to the space charge limited current (SCLC) model, the trap density is mainly determined by the trap‐filled‐limit voltage (*V*_TFL_).^[^ ^47^ ^]^ As can be seen, both modified devices exhibited a reduced *V*_TFL_ compared to the control device, suggesting a lower trap density for these devices.^[^ ^48^ ^]^ This result is in line with the passivation effect of phosphine oxides mentioned earlier. On the other hand, while the lowest unoccupied molecular orbital (LUMO) level of PPF is comparable to that of TPBi, the LUMO level of PPT is lower than that of TPBi but higher than the conduction band minimum (CBM) of the perovskite layer, presenting a more favorable energy level arrangement that facilitates the electron injections from TPBi. This is evidenced by the electron‐only devices. As shown in Figure S7 (Supporting Information), the electron‐dominated current density of PPT/PPF‐modified devices increases significantly, demonstrating the facilitated electron transport.

Figure 3c displays the current density–voltage–luminance (*J*–*V*–*L*) characteristics of the fabricated devices based on the pristine, PPT‐ and PPF‐modified perovskite films (named as the control, PPT and PPF devices, respectively). The best performance for each device is summarized and compared in **Table** **1**. It can be clearly seen that the PPT and PPF devices are more than twice as bright as the control device, reaching 73 896.6 and 61025.3 cd m^−2^ respectively, with a maximum external quantum efficiency (EQE) of more than 10%. To the best of our knowledge, this result, and in particular the brightness, is at the top tier of pure Cs‐based quasi‐2D PeLEDs. Such an improvement can be attributed to the facilitated electron transport and better hole‐blocking effect exerted by PPT/PPF, as discussed earlier. Due to the benefit of improved electron injection, the PTT and PPF devices also exhibit lower turn‐on voltages compared to the control device. Note that the EL peaks of the PPT and PPF device are almost identical to that of the control device (Figure S8, Supporting Information), which is consistent with the PL results.

**Table 1:** Performance of the fabricated PeLEDs

	*V*_on_ [V]	EL peak [nm]	Max. Lum. [cd m^−2^]	Max. EQE [%]
Control	3.0	512	26 471.2	14.5
PPT	2.8	513	73 896.6	10.7
PPF	2.8	512	61 025.3	12.6

In general, quasi‐2D perovskites have lower conductivity and slower carrier injection rates than pure 3D perovskites, resulting in a lower brightness than 3D perovskites.^[^ ^29^ ^]^ In addition, Joule heating and leaking carriers in the device lead to an abrupt decrease in EQE at high voltages, which is the main cause of poor performance and severe efficiency roll‐off.^[^ ^30^, ^49^, ^50^ ^]^ However, as shown in Figure 3d–f, the efficiency roll‐off at high voltages is significantly suppressed in PPT and PPF devices. This improvement is clearly due to the greater hole‐blocking effect exerted by PPT/PPF. A more detailed discussion of the improved brightness and efficiency roll‐off will be discussed in the following sections.

### 2.4 Investigation of the Recombination Dynamics by TA Measurement

**Figure 4:**
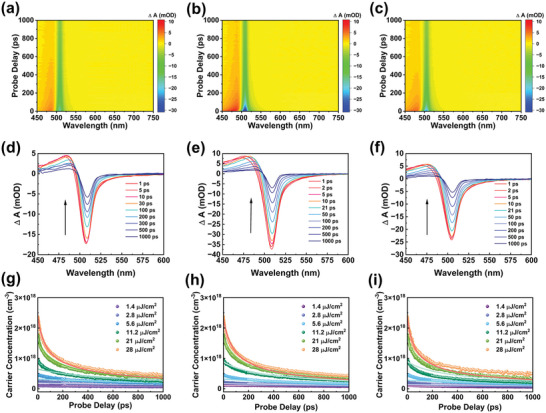
Color plot for transient absorption (TA) in the visible regions for a) pristine, b) PPT‐modified, and c) PPF‐modified perovskite films. Ultrafast time‐resolved TA spectra of d) pristine, e) PPT‐modified, and f) PPF‐modified perovskite films. Power‐dependent carrier dynamics at the probe wavelength of 505 nm for g) pristine, h) PPT‐modified, and i) PPF‐modified perovskite films.

To further explore the mechanism for the improved brightness and suppressed efficiency roll‐off, transient absorption (TA) spectroscopy was performed to understand the relaxation process of photoexcited carriers in the perovskite layer. As shown in **Figure** **4**a–c, the TA spectra show only one ground state bleaching (GSB) signal at 505 nm for all perovskite films, indicating the fact that the other quasi‐2D phases are considerably suppressed.^[^ ^15^ ^]^ Note that a band larger than 505 nm is attributed to the trap‐assisted nonradiative recombination, and the photocarriers are generated within a femtosecond timescale (<1 ps) at 505 nm (see Figure S9a–c, Supporting Information).^[^ ^51^ ^]^ This result verifies the existence of an exclusively dominant domain in the solution‐processed perovskite films, which is consistent with the steady‐state absorption spectra (Figure 1b).^[^ ^52^ ^]^

Afterward, the photocarriers in GSB gradually disappear and no other peaks are formed (Figure 4d–f), indicating that the carriers are finely confined in the quantum well structure and successfully recombine with each other. Notably, the change in transmittance of the modified films is greater than that of the control film, suggesting that more carriers are located at the perovskite band edge and excited by the laser beam after the PPT/PPF modification. Nevertheless, the absorption dynamic profiles before and after post‐modification are not significantly different, suggesting that the relaxation process of photoexcited carriers within the perovskite layer is not strongly affected by the PPT/PPF modification and that the increase in device brightness as well as the decrease in efficiency roll‐off may not be due to the difference in energy transfer between multiple quasi‐2D phases in the perovskite film.

We are therefore curious about the carrier dynamics of the perovskite emitting layer, as it has been widely reported that efficiency roll‐off is related to this.^[^ ^53^, ^54^ ^]^ In general, the recombination pathways of free carriers are divided into three routes: monomolecular trap‐assisted recombination, bimolecular radiative recombination, and trimolecular Auger recombination, in the sequence of the number of carriers involved.^[^ ^5^ ^]^ For metal halide perovskite materials, trap‐assisted and Auger recombination are classified as non‐radiative recombination. This suggests that the energy released through these two processes does not produce photons. Furthermore, as Auger recombination involves more carriers than other processes, it usually becomes more prominent at higher voltages. Therefore, the competition between radiative recombination and non‐radiative Auger recombination strongly affects the maximum EQE, brightness, and efficiency roll‐off of PeLEDs. The recombination pathway can be described as the following rate equation:^[^ ^5^ ^]^

(1) $$-\;\frac{dn}{dt}=k_{1}\;n+k_{2}n^{2}+k_{3}n^{3}$$

where *n* is the free charge carrier density and *k*_1_, *k*_2_, and *k*_3_ represent the coefficients for monomolecular trap‐assisted recombination, bimolecular radiative recombination, and trimolecular Auger recombination, respectively. In order to elucidate the relation between recombination dynamics and improved device performance, fluence‐dependent TA measurements were performed to obtain values for these coefficients.

Since the recombination dynamics depend strongly on the carrier concentration as shown in Equation (1), the initial carrier concentration is crucial for fluence‐dependent TA analysis. Therefore, we must measure the recombination dynamics at different initial carrier concentrations to obtain a more accurate recombination profile, which is achieved by applying multiple excitation beams with varied pump powers. In addition, it is not sufficient to extract *k*_1_ purely from the TA characterization, as the time scale of the TA spectroscopy does not exceed a few nanoseconds. Thus, *k*_1_ in Equation (1) was determined by fitting the TRPL data using a single exponential fit.^[^ ^53^ ^]^ The profiles of the carrier dynamics and the fitting results are shown in Figure 4g–i and are summarized in **Table** **2**. More details on the fluence‐dependent TA analysis can be found in the literature.^[^ ^53^, ^54^ ^]^ In summary, the global fit data show an increase in bimolecular recombination constants after post‐modification, which may be due to more excited carriers after the PPT/PPF modification, as we mentioned earlier. This trend is consistent with the prolonged average carrier lifetime in TRPL and the reduced *V*_TFL_ in SCLC, again confirming the passivation effect of PPT/PPF. However, to our surprise, the values of the Auger recombination constant did not show a noticeable decrease after post‐modification. This not only implies that the PPT/PPF modification did not succeed in reducing Auger recombination within the perovskite films, but also suggests that the reduced efficiency roll‐off of the modified devices did not originate from the inhibition of Auger recombination. Therefore, the cause of the reduced efficiency roll‐off remain unclear.

**Table 2:** Recombination decay constants of the control, PPT‐modified and PPF‐modified perovskite films

	*k*_1_ [s^−1^]	*k*_2_ [cm^3^s^−1^]	*k*_3_ [cm^6^s^−1^]
Control	1.5 × 10^8^	8.0 × 10^−10^	2.0 × 10^−27^
PPT	1.4 × 10^8^	1.8 × 10^−10^	1.3 × 10^−27^
PPF	1.2 × 10^8^	1.3 × 10^−10^	2.2 × 10^−27^

### 2.5 Investigation of the Carrier Dynamics between Interfaces in PeLED Device

**Figure 5:**
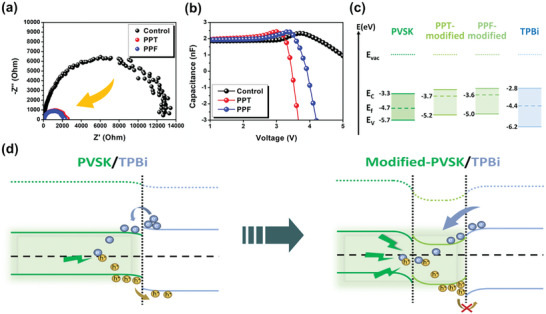
a) EIS analyses and b) capacitance‐voltage curves of control, PPT‐modified, and PPF‐modified devices. c) Energy levels of the pristine, PPT‐modified, PPF‐modified perovskite films, and TPBi. d) Schematic representation of the better carrier dynamics in the modified device.

Given that the results of the recombination dynamics cannot fully explain the improved performance and reduced efficiency roll‐off, we turned to the carrier dynamics within the whole device. In order to understand the electrical characteristics of the fabricated PeLEDs, we performed electrochemical impedance spectroscopy (EIS) measurements at 4 V, as shown in **Figure** **5**a. The modified PeLEDs have a much smaller arc radius in the Nyquist plot, which verifies that better electron injection and carrier transport capability are achieved with the introduction of the PPT/PPF layer.^[^ ^55^, ^56^, ^57^ ^]^ Furthermore, to better elucidate the carrier dynamics, capacitance‐voltage (*C*–*V*) measurements were conducted and the results are shown in Figure 5b. Due to the dielectric property of perovskite, PeLED is considered as a capacitor.^[^ ^58^, ^59^ ^]^ Therefore, we are able to monitor the carrier dynamics within the device by the capacitance measured at different applied voltages.

At the beginning, the number of carriers within the device is very small and negligible, so the capacitance of the device can be thought of as its geometric capacitance, which is a constant. As the applied voltage slowly increases, carriers begin to be injected into the device; however, the number of carriers that can be injected into the device is limited as the applied bias voltage is not large enough to overcome the energy barrier between each layer, resulting in a slow increase in capacitance. When the applied voltage is large enough, more and more carriers overcome the energy barrier and flow into the device, resulting in a phenomenon where the capacitance increases rapidly as the applied voltage increases. However, when the applied voltage exceeds the turn‐on voltage, a considerable number of carriers start to recombine with each other in the perovskite layer. This significantly reduces the number of carriers in the device and thereby reduces the capacitance value. In this regard, the competition between the number of carriers injected by increasing the applied voltage and the carriers eliminated by recombination eventually determines the capacitance after the turn‐on voltage. If the recombination rate is fast enough, the capacitance of the device will keep decreasing. In other words, the better the carriers are recombined in the perovskite layer, the lower the capacitance will be.^[^ ^60^ ^]^

Back to the experimental results illustrated in Figure 5b, it is not until 3 V that the capacitance of the control device begins to increase, while the capacitance of the modified devices increases slowly at the start. This indicates that carriers are more readily injected into the modified devices, where the overall energy barrier is smaller. This is a consequence of the improved energy level alignment resulting from the introduction of the PPT/PPF layer. Furthermore, the capacitance of the PPT/PPF‐modified devices starts to decrease at 3.0 and 3.5 V, respectively, whereas the capacitance of the control device starts to decrease at a higher voltage of ≈3.8 V. Together with the reduction in turn‐on voltage observed in the PPT/PPF‐modified devices, it reaffirms the improved energy barrier in the PPT/PPF‐modified devices. More importantly, the capacitance of the modified devices decreases rapidly after the turn‐on voltage, while the capacitance of the control device declines slowly. This suggests that the recombination behavior improves after the introduction of an additional PPT/PPF layer.^[^ ^58^, ^60^ ^]^ This improvement certainly stems from the hole‐blocking capabilities of PPT and PPF. Generally, holes start to escape from the perovskite layer when the energy gained per carrier is greater than the HOMO difference between perovskite and TPBi. This leads to a poor recombination behavior of the control device at high voltages. In contrast, since the potential barrier between perovskite and ETL is much larger in modified devices, electrons and holes can successfully recombine in perovskite layer regardless of the applied bias voltage.

More specifically, energy levels of the perovskite films without and with PPT/PPF modification were calculated. Figure 5c shows the energy levels of the perovskite films including CBM, valence band minimum (VBM), and Fermi levels, which were calculated from ultraviolet photoelectron spectroscopy (UPS) and UV–Vis absorption.^[^ ^61^ ^]^ Figure S10 (Supporting Information) shows the UPS results. As the layers of PPT and PPF are too thin to be measured by UPS, we can only obtain the energy levels of the perovskite surfaces modified by PPT and PPF. Moreover, it can be noted from the previous discussion that the bulk of the perovskite layer is almost unaffected by the surface modification. Therefore, we assume that the energy levels of the modified perovskite layers are the same as that of the control film, except for the interface with TPBi. Since the Fermi level of each layer is the same in equilibrium, the band edges will bend to achieve a continuous vacuum level.^[^ ^62^ ^]^ Initially, the energy levels of TPBi exhibited unfavorable upward band bending, while the energy levels of the perovskite layer also exhibited unfavorable downward bending at the interface between perovskite and TPBi, resulting in a large energy barrier to electron injection and poor hole blocking, as in the scheme shown on the left in Figure 5d. Fortunately, the energy level alignment becomes more favorable after the introduction of the PPT/PPF layer, as shown on the right of Figure 5d. The energy levels of PPT‐ and PPF‐modified perovskite films show not only a downward bending of the TPBi band but also an upward bending of the band edge of the surface perovskite. This facilitates the injection of electrons from TPBi into the perovskite layer while preventing the escape of holes from the emitting layer.^[^ ^63^ ^]^

This result is consistent with other analyses mentioned above. As a result of the PPT/PPF modification, the energy barrier for electron injection becomes smaller, making it easier for carriers to be injected into the perovskite layer, thereby increasing the current density and reducing the turn‐on voltage. At the same time, the better hole‐blocking capability in the modified devices also make it easier for electrons and holes to recombine at high applied bias voltages, leading to increased brightness and EQE at high voltages. More importantly, by introducing an additional PPT/PPF layer, the efficiency roll‐off is greatly suppressed, showing a straightforward and effective way to deal with one of the most challenging problems for quasi‐2D PeLEDs. However, despite the ability of PPT and PPF to successfully passivate defects on the perovskite surface, as demonstrated in XPS and SCLC measurements, the maximum EQEs of the modified devices did not exceed the value of the control device. This still requires more detailed analyses to elucidate the reasons behind this.

## 3 Conclusion

In summary, we describe a simple and effective method to reduce the efficiency roll‐off of PeLEDs through interface engineering. By introducing a thin layer of conductive phosphine oxides, PPT or PPF, at the perovskite/ETL interface, surface defects on the perovskite layer are not only passivated, but the carrier dynamics across the interface are also improved. As a result of the facilitated electron injection and enhanced hole‐blocking effect, the PPT/‐PPF modified devices show much higher brightness and much lower efficiency roll‐off at high bias voltages than the control device, while the performance of these device is also remarkable among those made from the same composition. Notably, our analysis shows that the improved device performance is mainly due to improved carrier dynamics at the perovskite/ETL interface rather than improved energy transfer between multiple quasi‐2D phases in the perovskite film. This result highlights the importance of the perovskite interface for device's efficiency roll‐off and provides a new perspective for constructing reliable high‐performance PeLEDs.

## 4 Experimental Section

### Materials

Lead bromide (PbBr_2_, >99.9%) and 2,8‐Bis(diphenyl‐phosphoryl)‐dibenzo[b,d]furan (PPF, ≥96%) were purchased from Tokyo Chemical Industry Co., Ltd. Cesium bromide (CsBr, >99.9%), phenethylammonium bromide (PEABr, ≥98%), 2,8‐Bis(diphenyl‐phosphoryl)‐dibenzo[b,d]thiophene (PPT, >96.5%), Poly[N,N″‐bis(4‐butylphenyl)‐N,N″‐bis(phenyl)‐benzidine] (Poly‐TPD), Poly(9‐vinylcarbazole) (PVK), lithium fluoride (LiF, >99.99%), and solvents including chlorobenzene (CB, 99.8%) and dimethyl sulfoxide (DMSO, ≥99.9%) were purchased from Sigma‐Aldrich and used without further purification. 2,2′,2″‐(1,3,5‐Benzinetriyl)‐tris(1‐phenyl‐1‐Hbenzimidazole) (TPBi) was purchased from Ultra Fine Chemical Technology Corp. 1,4,7,10,13,16‐Hexaoxacyclooctadecane (18‐crown‐6, 99%) was purchased from Alfa Aesar.

### Device Fabrication

Precursor solution of quasi‐2D perovskite was prepared by dissolving 16.2 mg PEABr, 42.6 mg CsBr, and 73.4 mg PbBr_2_ in 1 mL of DMSO with 3.5 mg 18‐crown‐6 and the solution was stirred at room temperature overnight. The solutions of PPT/PPF were prepared by dissolving 2 mg PPT/PPF in 1 mL of CB. The ITO and glass substrates were first cleaned with detergent and then sonicated in deionized water, acetone, and isopropyl alcohol in successive steps of 15 min each. They were finally placed in an oven at 60 °C to remove residual solvents. The cleaned substrates were treated using a plasma cleaner prior to device fabrication and then transferred into a N_2_‐filled glovebox. The Poly‐TPD and PVK layers were spin‐coated onto the substrate at 2000 rpm for 60 and 30 s, respectively, and then annealed at 150 °C. Afterward, the quasi‐2D perovskite film was spin‐coated onto the bilayer HTL at 1000 rpm for 5 s and 3000 rpm for 55 s and then annealed at 100 °C for 60 s. After soaking in the precursor solution for 30 s, a thin layer of PPT or PPF was spin‐coated onto the perovskite surface at 5000 rpm for 60 s. Finally, the device was completed by continuous vacuum deposition of TPBi, LiF, and Al in a high vacuum environment (<10^−6^ Torr) with thicknesses of 25, 1, and 100 nm for TPBi, LiF, and Al, respectively.

### Characterization

The quasi‐2D perovskite films used for characterization were prepared in the same method as described above. Absorption spectra were recorded using a Jasco V‐730 UV−Visible spectrophotometer system and the emission spectra were measured using a Horiba Fluorolog‐3 spectrometer system. Time‐resolved photoluminescence (TRPL) measurements were carried out using the Hamamatsu Universal Streak Camera C10910 at the National Synchrotron Radiation Research Center (NSRRC) of TPS 23A section. The X‐ray diffraction (XRD) patterns were studied by Rigaku SmartLab SE. The SEM images of the films were taken with a Hitachi S4800 machine. The nuclear magnetic resonance (NMR) spectra were tested on Bruker AVIII‐500 MHz FT‐NMR spectrometer. The optimized configurations of PPT and PPF were determined by Gaussian 09 W with density functional theory (DFT) calculation. The B3LYP method with 6–311G(*d*,*p*) basic set was applied to the ground‐state molecular simulation. PLQY and electrical characterizations, including space‐charge‐limited current (SCLC), current density–voltage–luminance (*J*–*V*–*L*), external quantum efficiency (EQE), and electroluminescence (EL) characteristics, were recorded by an LQ‐100 measuring system from Enlitech Co. Ltd with a 4 mm^2^ integrating sphere. Measurements were conducted in the N_2_‐filled glovebox and the devices were not encapsulated.

A commercial transient absorption spectrometer (Femto Frame II, IB Photonics) was used to study the carrier dynamics of perovskite films. For pump‐probe transient absorption spectroscopy measurements, the excitation source was a 400 nm pulsed laser with a pulsed width of 100 fs and a repetition rate of 1 kHz, which was generated by an optical parameter amplifier (OPA, TOPAS‐C, Light Conversion) system with a mode‐locked Ti:Sapphire laser (Spectra‐Physics, Tsunami). Both the pump beam and the probe beam were focused on the sample through different lenses; the focal spot sizes of the pump and probe beams were 300 and 100 µm in diameter, respectively. The 400 nm pump laser beam was generated using an OPA system, while the broadband (450‒800 nm) white‐light probe beam with a pulse width of 30 fs was generated by supercontinuum generation from a thick sapphire plate. Pump densities range from ≈1.4 to 28 µJ cm^−2^. All optical measurements were performed under ambient conditions with humidity below 50%. X‐ray photoelectron spectroscopy (XPS) and ultraviolet photoelectron spectroscopy (UPS) measurements were carried out by PerkinElmer PHI 5400 equipped with a monochromatized Al K*α* X‐ray photon discharge lamp and specs UVS 10/35, respectively. The HOMO energy levels and *E_g_* s of the materials were calculated from their Tauc plots under the assumption of direct *E_g_*. Electrochemical impedance spectroscopy (EIS) and capacitance‐voltage curves were measured in air using a BioLogic SP‐200 Impedance/Frequency response analyzer on encapsulated devices with AC impedance spectroscopy

## Conflict of Interest

The authors declare no conflict of interest.

## Supporting information

Supporting Information

## Data Availability

The data that support the findings of this study are available from the corresponding author upon reasonable request.
