# Supplementary material for: Realizing High Brightness Quasi‐2D Perovskite Light‐Emitting Diodes with Reduced Efficiency Roll‐Off via Multifunctional Interface Engineering
Source: Adv Sci (Weinh). 2023 Jul 3;10(26):2302232. doi: 10.1002/advs.202302232 (PMC10502845; doi:10.1002/advs.202302232)
Supplement: Supplementary file 1 — Supporting Information [file ADVS-10-2302232-s001.pdf]

ADVANCED SCIENCE
Open Access

Supporting Information

for *Adv. Sci.*, DOI 10.1002/advs.202302232

Realizing High Brightness Quasi-2D Perovskite Light-Emitting Diodes with Reduced Efficiency Roll-Off via Multifunctional Interface Engineering

Yu-Kuan Lin, Chiung-Han Chen, Yen-Yu Wang, Ming-Hsuan Yu, Jing-Wei Yang, I-Chih Ni, Bi-Hsuan Lin, Ivan S. Zhidkov, Ernst Z. Kurmaev, Yu-Jung Lu and Chu-Chen Chueh**

Supporting Information

Realizing High Brightness Quasi-2D Perovskite Light-Emitting Diodes with Reduced Efficiency Roll-Off via Multifunctional Interface Engineering

Yu-Kuan Lin,[a] *Chiung-Han Chen,*[a] *Yen-Yu Wang,*[b] *Ming-Hsuan Yu,*[a] *Jing-Wei Yang,*[b] *I-Chih Ni,*[c] *Bi-Hsuan Lin,*[d] *Ivan S. Zhidkov,*[e,f] *Ernst Z. Kurmaev,*[e,f] *Yu-Jung Lu*[b,g]* *and Chu-Chen Chueh*[a,h]*

[a] Y.-K. Lin, C.-H. Chen, M.-H. Yu and Prof. C.-C. Chueh
Department of Chemical Engineering, National Taiwan University, Taipei 10617, Taiwan

[b] Y.-Y. Wang, J.-W. Yang and Prof. Y.-J. Lu
Research Center for Applied Sciences, Academia Sinica, Taipei 11529, Taiwan

[c] I-C. Ni
Graduate Institute of Photonics and Optoelectronics, National Taiwan University, Taipei 10617, Taiwan

[d] Dr. B.-H. Lin
National Synchrotron Radiation Research Center, Hsinchu 30076, Taiwan

[e] Prof. I. S. Zhidkov and Prof. E. Z. Kurmaev,
Institute of Physics and Technology, Ural Federal University, Yekaterinburg 620002, Russia

[f] Prof. I. S. Zhidkov and Prof. E. Z. Kurmaev
M. N. Mikheev Institute of Metal Physics of Ural Branch of Russian Academy of Sciences, Yekaterinburg 620108, Russia

[g] Prof. Y.-J. Lu
Department of Physics, National Taiwan University, Taipei 10617, Taiwan

[h] Prof. C.-C. Chueh
Advanced Research Center for Green Materials Science and Technology, National Taiwan University, Taipei 10617, Taiwan

*Corresponding author. E-mail: cchueh@ntu.edu.tw; yujunglu@ gate.sinica.edu.tw

Keywords: Light-emitting diodes; quasi-2D perovskite; efficiency roll-off; interface engineering; hole blocking

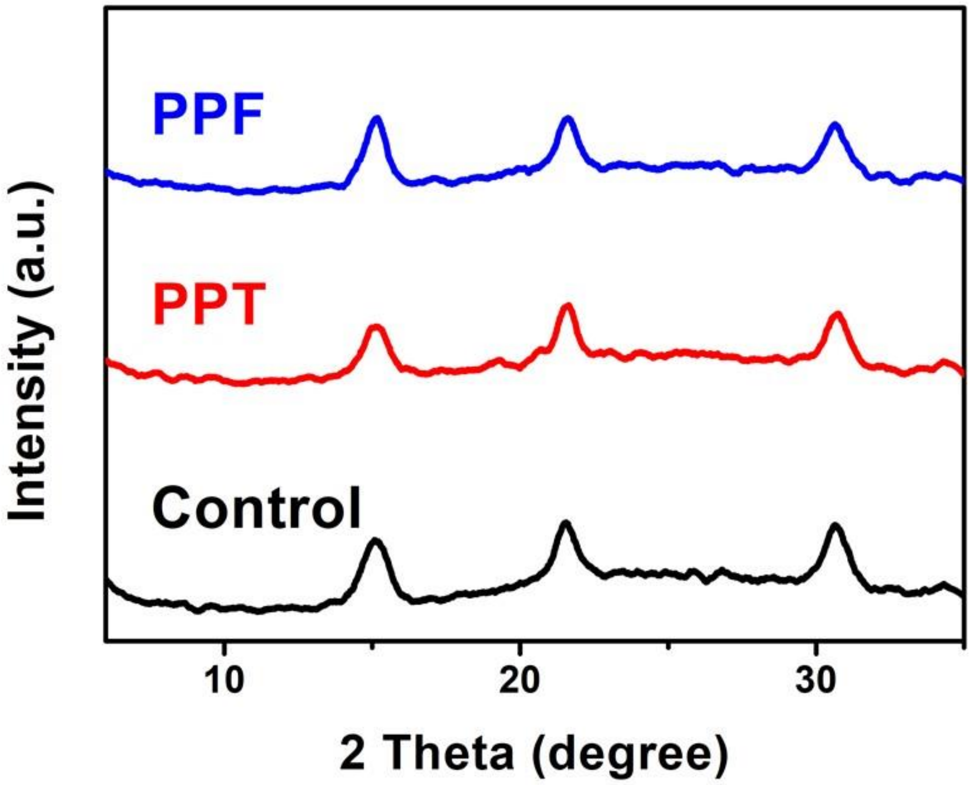

Figure S1. XRD characteristics of the pristine, PPT-modified and PPF-modified perovskite films.

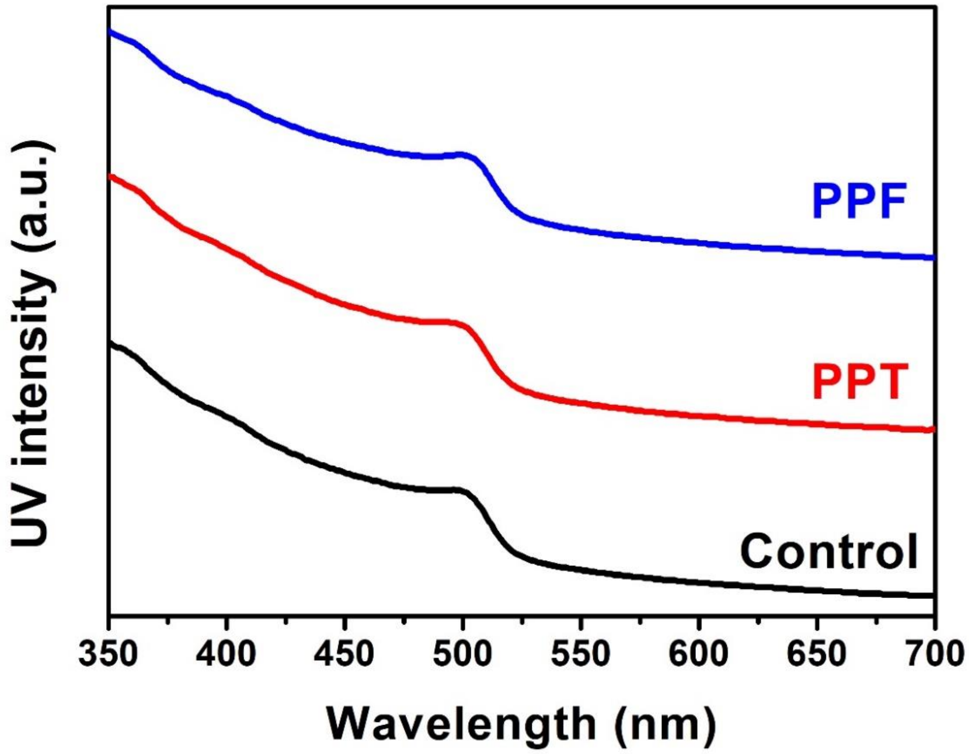

Figure S2. UV−vis absorption spectra of the pristine, PPT-modified and PPF-modified perovskite films.

Table S1. Biexponential fitting parameters for the TRPL results.

	A_1	τ_1 (ns)	A_2	τ_2 (ns)	τ_{avg} (ns)
**Control**	4.44×10^4	6.26	4.88×10^3	45.5	10.1
**PPT**	3.67×10^4	6.83	4.33×10^3	57.4	12.2
**PPF**	1.90×10^4	8.21	3.82×10^3	67.6	18.2

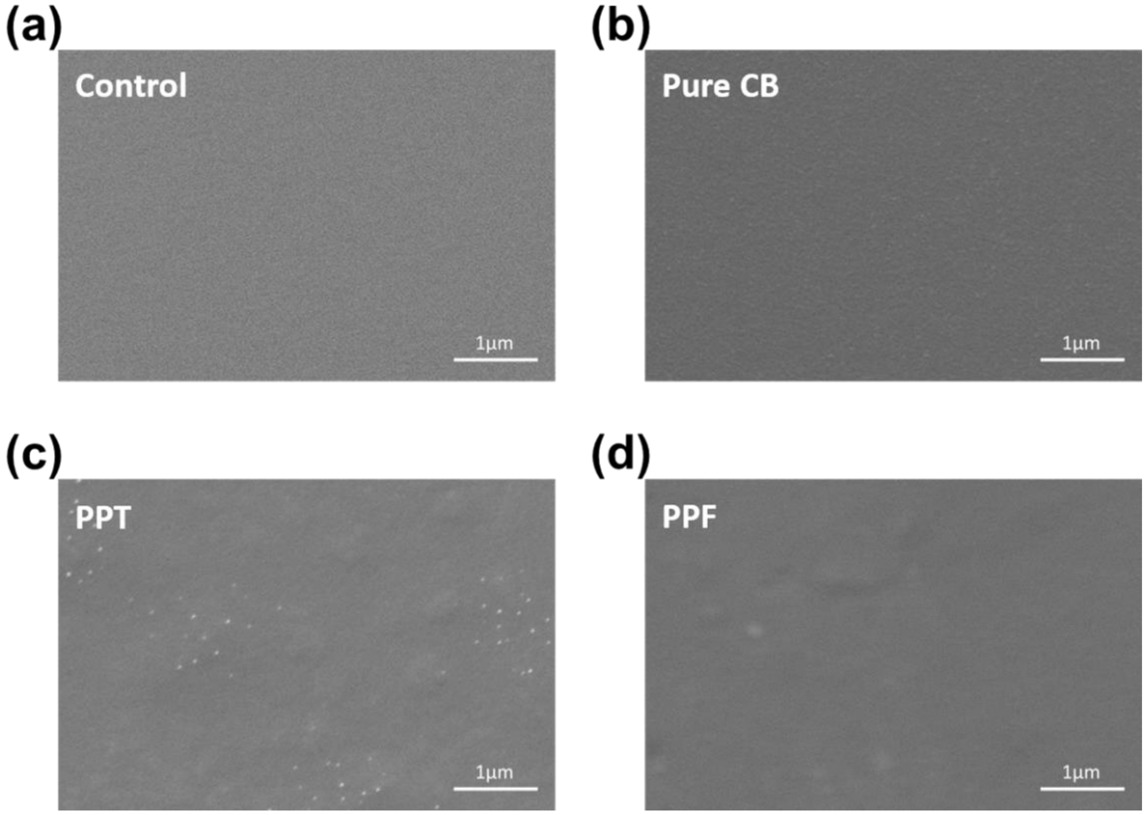

Figure S3. SEM images of the (a) pristine, (b) CB-treated, (c) PPT-modified and (d) PPF-modified perovskite films.

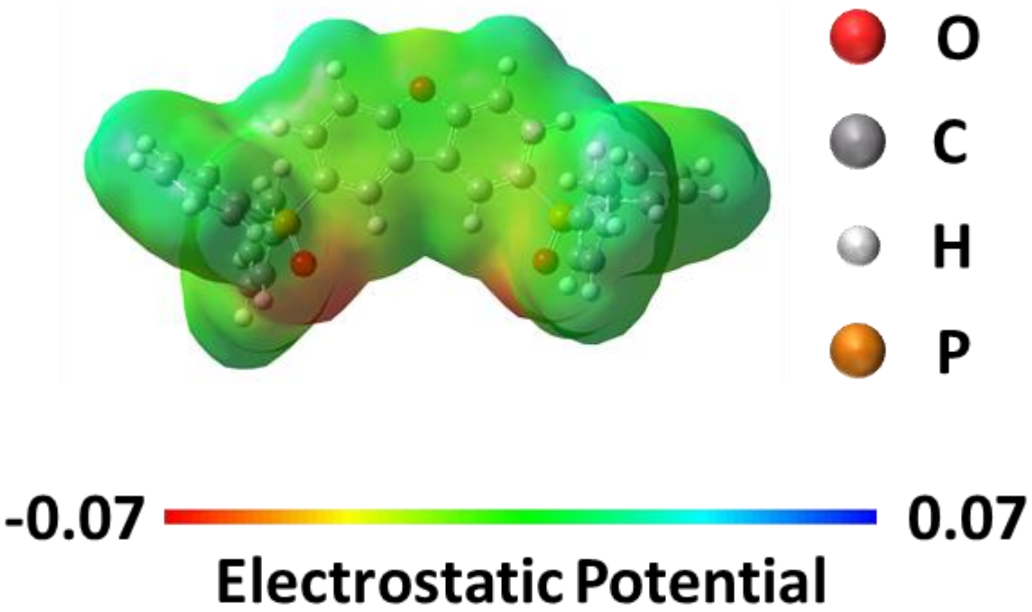

Figure S4. ESP map of PPF molecule.

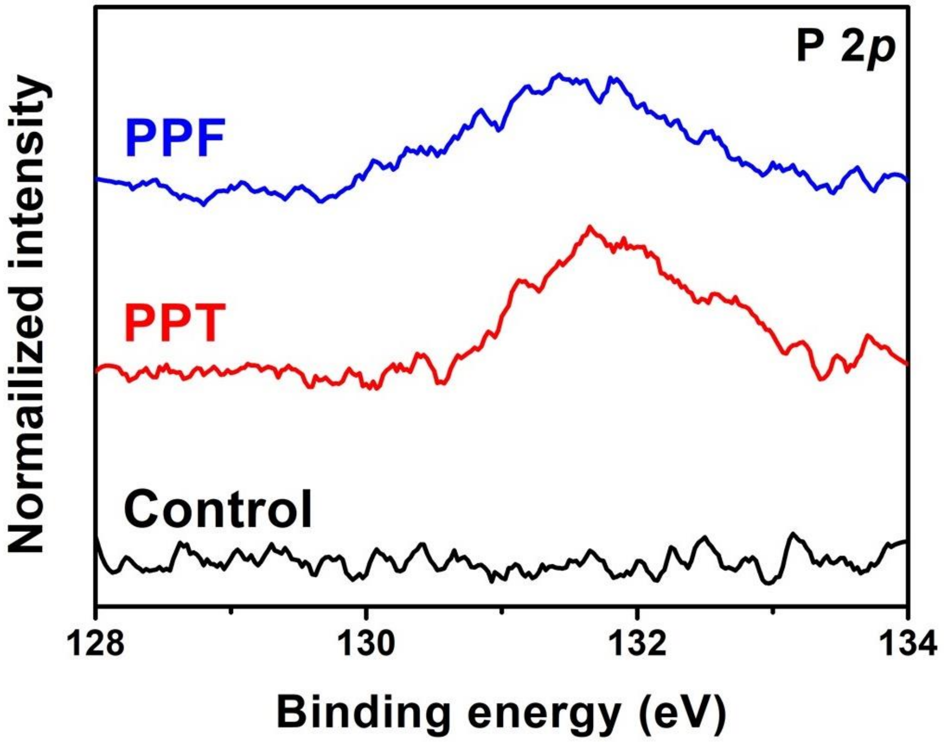

Figure S5. XPS spectra of P 2p signal for the pristine, PPT-modified and PPF-modified perovskite films.

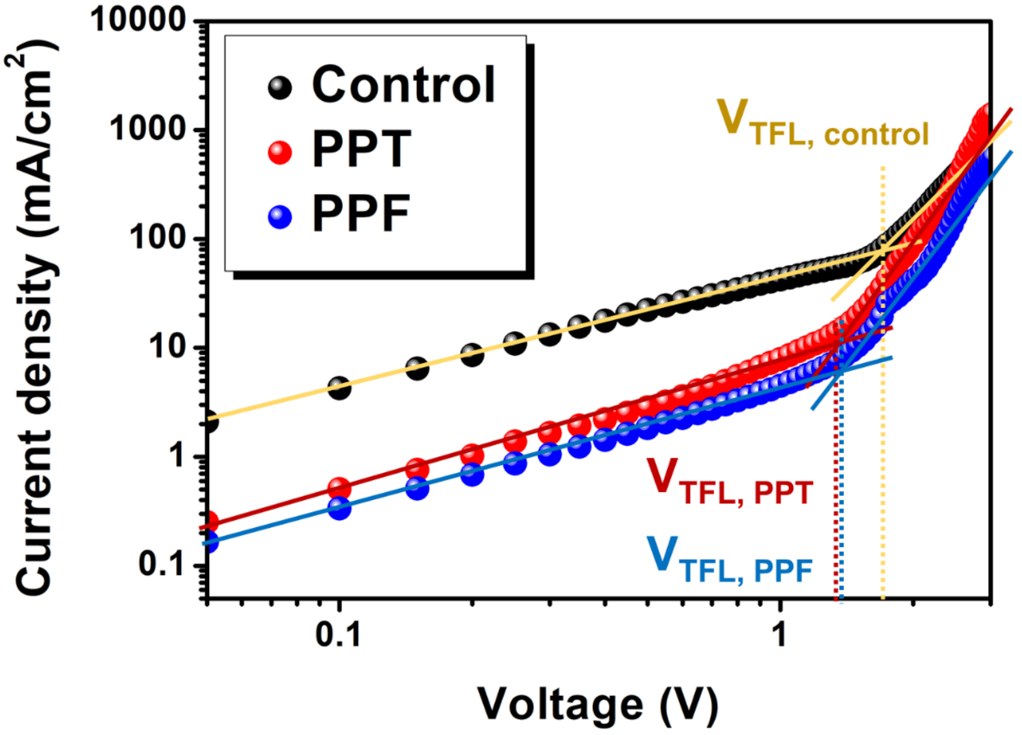

Figure S6. *J-V* characteristics of the studied hole-only devices.

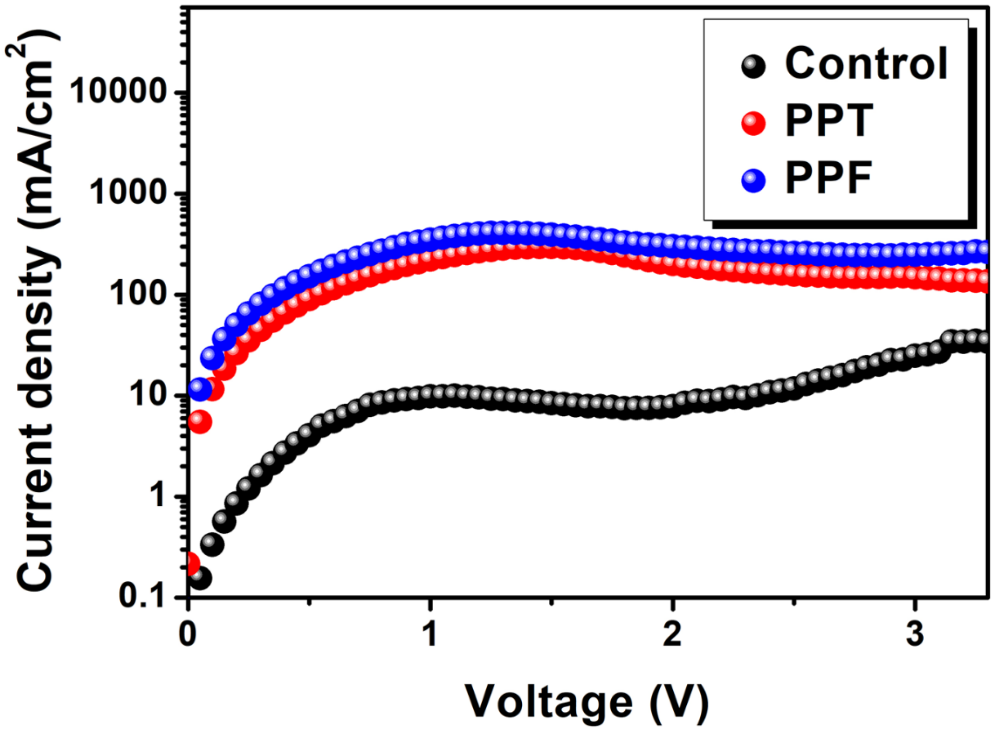

Figure S7. *J-V* characteristics of the studied electron-only devices.

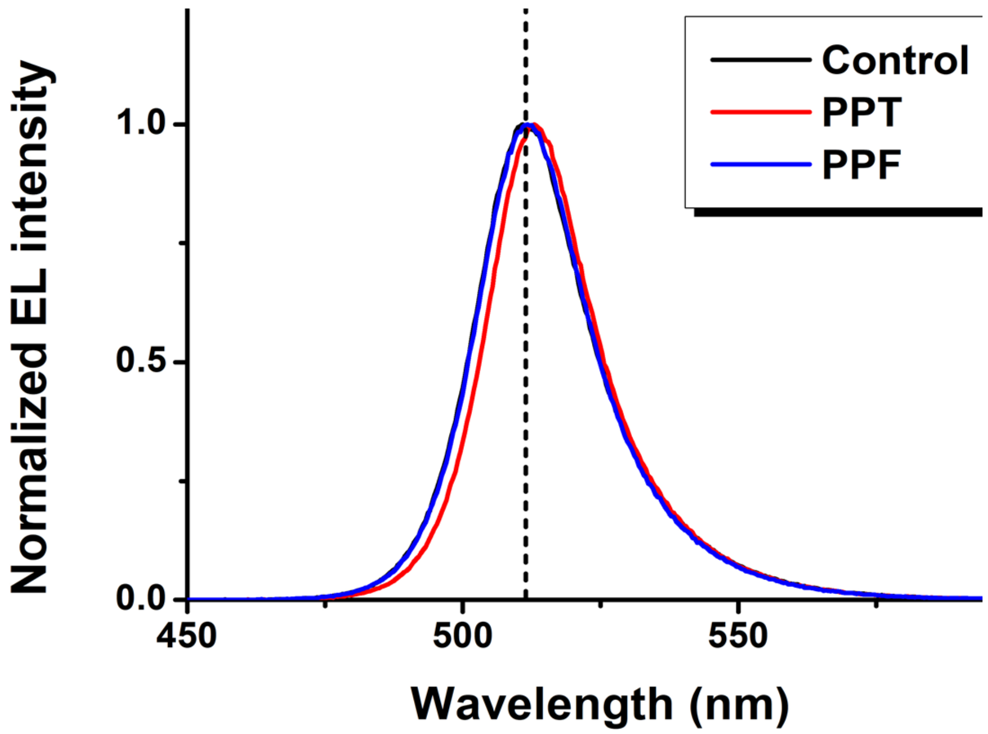

Figure S8. EL curves of the fabricated PeLEDs measured at 4V.

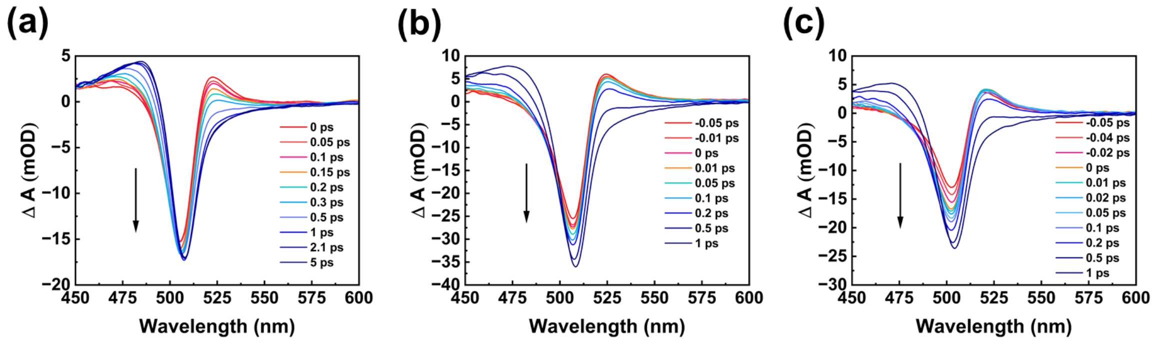

Figure S9. Femtosecond time-resolved TA spectra of (a) pristine, (b) PPT-modified and (c) PPF-modified perovskite films.

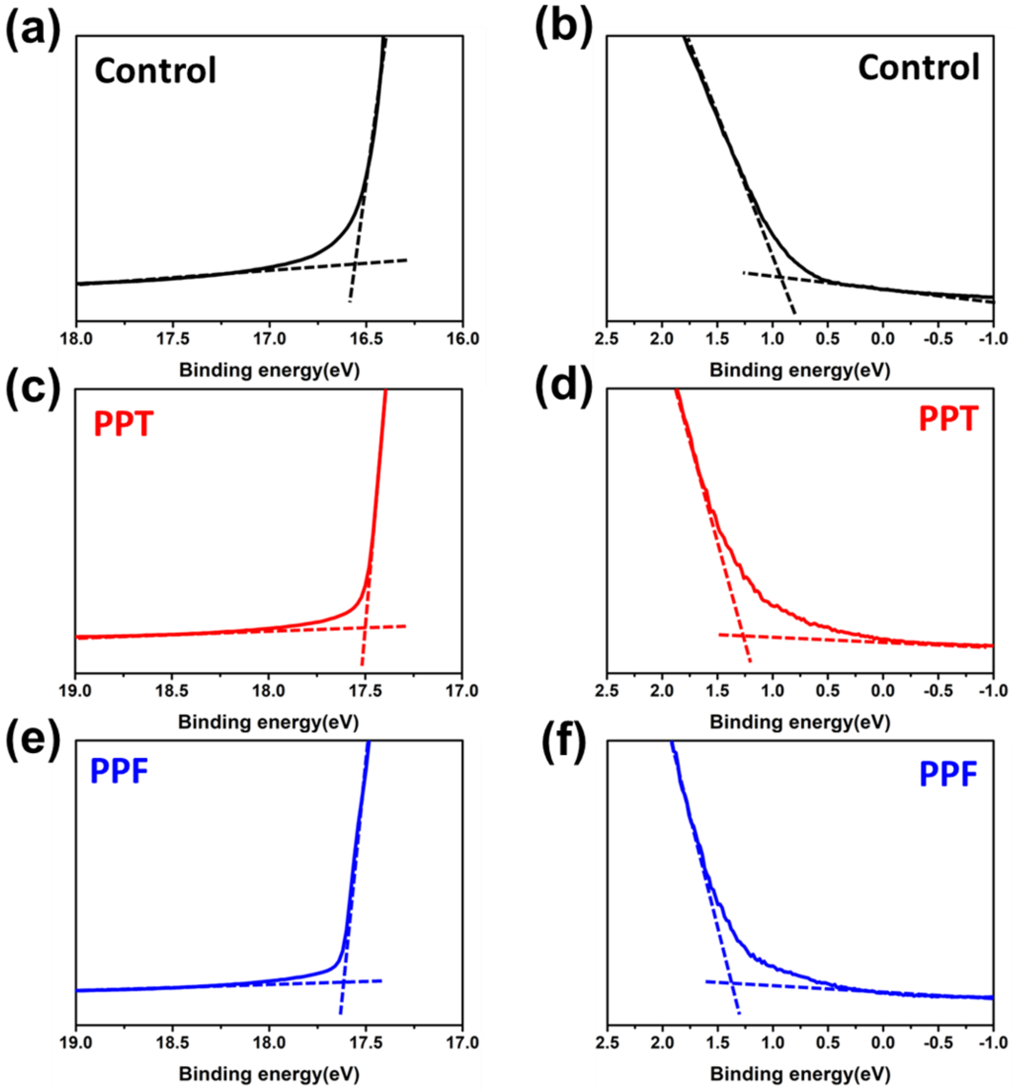

Figure S10. UPS results of the (a,b) pristine, (c,d) PPT-modified and (e,f) PPF-modified perovskite films.
